# Deletion of the meq gene significantly decreases immunosuppression in chickens caused by pathogenic marek's disease virus

**DOI:** 10.1186/1743-422X-8-2

**Published:** 2011-01-05

**Authors:** Yanpeng Li, Aijun Sun, Shuai Su, Peng Zhao, Zhizhong Cui, Hongfei Zhu

**Affiliations:** 1 Institute of Animal Sciences, Chinese Academy of Agricultural Sciences, Beijing, 100193, PR China; 2 Animal Science and Technology College, Shandong Agricultural University, Tai'an, Shandong, 271018, PR China

## Abstract

**Background:**

Marek's disease virus (MDV) causes an acute lymphoproliferative disease in chickens, resulting in immunosuppression, which is considered to be an integral aspect of the pathogenesis of Marek's disease (MD). A recent study showed that deletion of the Meq gene resulted in loss of transformation of T-cells in chickens and a Meq-null virus, rMd5ΔMeq, could provide protection superior to CVI988/Rispens.

**Results:**

In the present study, to investigate whether the Meq-null virus could be a safe vaccine candidate, we constructed a Meq deletion strain, GX0101ΔMeq, by deleting both copies of the Meq gene from a pathogenic MDV, GX0101 strain, which was isolated in China. Pathogenesis experiments showed that the GX0101ΔMeq virus was fully attenuated in specific pathogen-free chickens because none of the infected chickens developed Marek's disease-associated lymphomas. The study also evaluated the effects of GX0101ΔMeq on the immune system in chickens after infection with GX0101ΔMeq virus. Immune system variables, including relative lymphoid organ weight, blood lymphocytes and antibody production following vaccination against AIV and NDV were used to assess the immune status of chickens. Experimental infection with GX0101ΔMeq showed that deletion of the Meq gene significantly decreased immunosuppression in chickens caused by pathogenic MDV.

**Conclusion:**

These findings suggested that the Meq gene played an important role not only in tumor formation but also in inducing immunosuppressive effects in MDV-infected chickens.

## Background

Marek's disease (MD) is a neoplastic disease of chickens, which is caused by the lymphotropic alphaherpesvirus, MD virus (MDV). MD is characterized by the development of T-cell lymphomas and lymphocytic infiltration of peripheral nerves, skin, skeletal muscle and visceral organs [1-3]. Infection with MDV and subsequent development of MD is frequently associated with immunosuppression, which is considered to be an integral aspect of MD pathogenesis that ultimately leads to the death of many chickens in a number of cases [4,5].

To search for oncogene(s), early studies focused on the genes expressed in tumor cells. It has been shown that the transcriptional activity of MDV in tumor cells was confined to the R_L _regions. And Meq [6], pp38 [7] and the BamHI-H family which includes a 132 bp repeating region [8-10] are unique to MDV among the R_L_-encoded genes. Inoculation of MD-susceptible birds with a pp38 deletion mutant virus revealed that pp38 is involved in early cytolytic infection of lymphocytes, but not the induction of tumors [11]. Recently studies showed that the mechanism of attenuation of MDV does not involve the 132 bp repeat region [12]. Among these genes, only Meq is the most consistently expressed in latent phase [6] which is present in serotype 1 strains, but not in the non-oncogenic serotype 2 and serotype 3 strains [13,14]. Meq is a 339 amino acid protein, characterized by a N-terminal bZIP domain which is closely related to the Jun/Fos oncoproteins and a proline-rich C-terminal transactivation domain [6]. Down-regulation of Meq resulting in the loss of the colony formation ability of MSB1 [15], over-expression of Meq resulting in the transformation of a rodent fibroblast cell line, Rat-2 [16], and the interaction between Meq and C-terminal-binding protein (CtBP), a highly conserved cellular transcriptional co-repressor [17], all suggests that Meq is likely to be one of the principal oncogene for MDV. The strongest evidence proving that Meq is an MDV oncogene was confirmed by Meq knockout experiments [18].

The direct relationship between MDV strains of higher pathogenicity and greater immunosuppression [4] suggest that Meq perhaps plays an important role in immunosuppression. In earlier studies we cloned the full length genome of the MDV strain, GX0101, into a bacterial artificial chromosome (BAC) and reconstituted the infectious virus, bac-GX0101 [19,20]. Studies in specific-pathogen-free (SPF) chickens showed that the virulence of bac-GX0101 could be classified from virulent to very virulent, and there was no difference in growth ability and pathogenicity to birds when compared with its parental virus, GX0101 [19]. In this report, we examined the oncogenic potential of GX0101ΔMeq, which was generated by deleting both copies of the Meq gene from bac-GX0101. Pathogenesis studies in SPF chickens showed that the MDV-encoded Meq gene is not only a principal oncogene but also involved in immunosuppression.

## Results

### Identification of Meq deletion mutant GX0101ΔMeq

**Figure 1:**
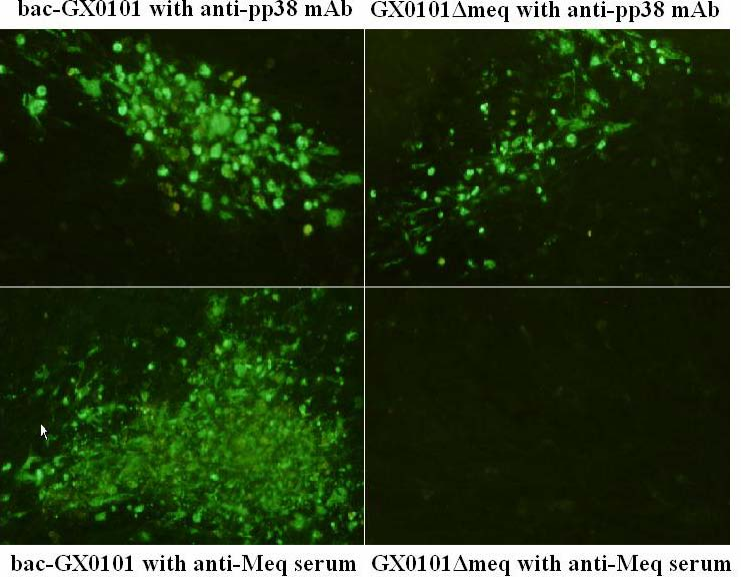
**Immunofluorescence analysis of CEF cells infected with recombinant viruses**. 100 PFU of bac-GX0101 and GX0101ΔMeq were inoculated into 6-well plates containing a monolayer of CEFs. The mAb H19 specific for the MDV-unique protein pp38, and mouse serum against Meq were used for immunofluorescence analysis. Parental virus, bac-GX0101 expressed Meq protein, whereas the deletion mutant virus GX0101ΔMeq did not. The presence of GX0101ΔMeq virus was confirmed by staining of MDV-specific pp38 protein.

Using BAC clones, we generated a mutant virus lacking both copies of the Meq gene, GX0101ΔMeq. Plaques from recombinant GX0101ΔMeq and control bac-GX0101 viruses were evident after five days following transfection. To confirm the deletion of the Meq gene, transfected cells showing plaques were examined by immunofluorescence assay (IFA) with monoclonal antibody (mAb) H19 and mouse anti-Meq polyclonal serum. As expected, bac-GX0101 virus expressed both pp38 and Meq, whereas GX0101ΔMeq expressed pp38 but not Meq (Figure 1).

### GX0101ΔMeq exhibited the same replication rate in CEF as bac-GX0101

To determine whether the deletion of the Meq gene has any effect on replication of GX0101ΔMeq in vitro, the growth rate of GX0101ΔMeq virus was compared with that of bac-GX0101. At hours 24, 48, 72, 96, 120 and 144 post-inoculation (p.i.), the recombinant virus GX0101ΔMeq exhibited the same replication dynamics in CEF as its parental virus bac-GX0101.

### Viremia levels of birds infected with GX0101ΔMeq or bac-GX0101

The viremia levels of birds infected with GX0101ΔMeq or bac-GX0101 were determined on days 7, 14, 21 p.i. As indicated in Table 1, with the exception of days 7 p.i., the viremia levels of GX0101ΔMeq virus-infected group were significantly lower than that of bac-GX0101 group on days 14 and 21 p.i.

**Table 1:** Comparision of viremia levels between bac-GX0101 and GX0101ΔMeq infected SPF chickens (n = 6)

Days post-infection	Viremia (PFU/ml)	
	
	bac-GX0101	GX0101ΔMeq
7	28 ± 9.8 a	32 ± 6.2 a
14	154 ± 47.6a	69.7 ± 16.5 b
21	256.5 ± 46.8 a	78.2 ± 9.5 b

All chickens were inoculated with 1000 PFU of the indicated virus by a subcutaneous route. The difference in vivo replication between bac-GX0101 and GX0101ΔMeq was determined by viremia levels. The numbers in the table indicate: mean ± standard deviation, at days 7, 14 and 21 p.i., and same letters indicate that the differences were not significant (*P *> 0.05), different letters indicate that a significant difference (*P *< 0.05).

### Pathogenicity of GX0101ΔMeq

**Figure 2:**
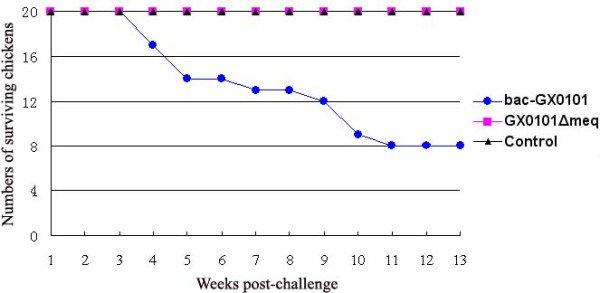
**Incidence of mortality in chickens inoculated with bac-GX0101 and GX0101ΔMeq**. Chickens were inoculated with 1000 PFU of the indicated viruses when they were one-day-old and maintained in isolation for 13 weeks. Mock-inoculated chickens served as negative controls and weekly mortality was recorded. Chickens that died during the experiment were evaluated for MDV-specific gross lesions.

To determine whether deletion of the Meq gene affects the pathogenic properties of MDV, chickens inoculated with bac-GX0101 or GX0101ΔMeq were observed for mortality for a period of 13 weeks. All chickens which died during the experiment or at termination were examined for MDV-specific lesions, including gross tumors and nerve lesions. As indicated in Figure 2 and Table 2 one chicken from GX0101ΔMeq group died on days 3 p.i. and two chickens from bac-GX0101 group died due to unidentified causes on days 8 p.i. MDV-associated mortality was observed in the parental bac-GX0101 group starting at three weeks after infection and only six chickens survived in the duration of the experiment. There was no MDV-associated mortality in mock- or GX0101ΔMeq-inoculated groups. All the chickens in the bac-GX0101 group had developed MDV-specific lesions, whereas none were observed in either GX0101ΔMeq- or mock-inoculated groups.

**Table 2:** The pathogenicity of bac-GX0101 and GX0101ΔMeq in chickens

Virus	Mortality due to MD (%)	MD-specific tumors (%)	MD-specific lesions (%)
bac-GX0101	60	25	100
GX0101ΔMeq	0	0	0
Control	0	0	0

All chickens were inoculated with 1000 PFU of the indicated virus by a subcutaneous route.

### Effect of GX0101ΔMeq on immune organ

Statistical analysis showed that the weights of the body, the relative thymus and bursa in bac-GX0101 group were significantly lower than the control group chickens and those infected with GX0101ΔMeq (*P *< 0.05) at 14 days p.i. There were no significant differences between chickens in the GX0101ΔMeq and control groups (*P *> 0.05; Table 3).

**Table 3:** Body weight and relative immune organs weight (n = 20)

Virus	Body weight (g)	Relative thymus weight (%)	Relative bursa weight (%)
bac-GX0101	91.9 ± 9.1 a	0.21 ± 0.06 a	0.2 ± 0.07 a
GX0101ΔMeq	116.6 ± 11.9 b	0.42 ± 0.08 b	0.38 ± 0.08 b
Control	125.9 ± 16.7 b	0.48 ± 0.09 b	0.45 ± 0.12 b

All chickens were inoculated with 1000 PFU of the indicated virus by a subcutaneous route. The numbers in the table indicate: mean ± standard deviation. Same letters indicate that the differences were not significant (*P *> 0.05), different letters indicate that a significant difference (*P *< 0.05).

**Figure 3:**
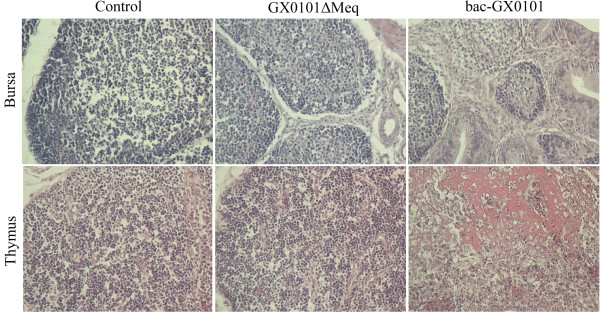
**Histological lesions with hematoxylin-eosin staining of bursa fabricii and thymus after inoculation with GX0101ΔMeq and bac-GX0101 at 400 × magnification**. At days 28 p.i. almost all of the chickens infected with bac-GX0101 presented with obvious atrophy of bursal follicles, displayed fibrous connective tissue hyperplasia, inflammatory exudate and necrotic cells infiltrated in the follicular interstitium, vague boundary among follicular cortex and follicular medulla, rarities of follicular cortex and loss of lymphocytes in the medullary area of bursal follicles. Thymus lesions lacking structure in the cortex and medulla, necrosis and disintegration of lymphocytes were also observed in birds infected with bac-GX0101. No MD-specific lesions were observed in the control and GX0101ΔMeq-infected groups.

The chickens infected with bac-GX0101 were grossly found to have typical atrophy of the bursa of Fabricius during the whole observation period. Overall, almost all of the chickens presented with obvious atrophy of bursal follicles, displayed fibrous connective tissue hyperplasia, inflammatory exudate and necrotic cells infiltrated in the follicular interstitium, vague boundary among follicular cortex and follicular medulla, rarities of follicular cortex and loss of lymphocytes in the medullary area of bursal follicles (Figure 3). Moreover, thymus lesions lacking structure in the cortex and medulla, necrosis and disintegration of lymphocytes were also observed in some birds infected with bac-GX0101 (Figure 3). No MD-specific lesions lesions were observed in the control and GX0101ΔMeq-infected groups.

### Effect of bac-GX0101 and GX0101ΔMeq on blood parameters

**Figure 4:**
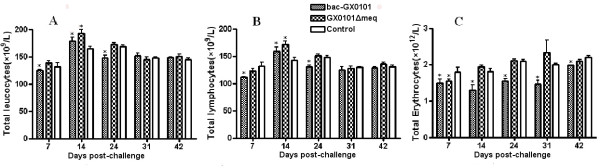
**Effects on some blood parameters of GX0101 and GX0101ΔMeq (n = 5)**. A: Total leucocytes; B: Total lymphocytes; C: Total erythrocytes. At 7, 14, 21, 31 and 42 days p.i., five chickens were randomly selected from each treatment. *P < 0.05 compared with those in control group. The means ± SD at each time point are shown. The number of leukocytes and lymphocytes in peripheral blood progressively increased in chickens infected with bac-GX0101 and GX0101ΔMeq compared with those in control group at days 14 p.i. And chickens displayed anemia with the number of erythrocytes progressively reduced on days 7, 14, 24, 31 and 42 after infection with bac-GX0101.

The number of leukocytes and lymphocytes in peripheral blood progressively increased in chickens infected with bac-GX0101 and GX0101ΔMeq compared with those in control group at days 14 p.i. (*P *< 0.05). There seems to be only minor variations in leukocyte and lymphocyte numbers among the three groups at days 24, 31 and 42 p.i. (*P *> 0.05, Figure 4A and 4B). Chickens displayed anemia with the number of erythrocytes progressively reduced on days 7, 14, 24, 31 and 42 after infection with bac-GX0101 (*P *< 0.05, Figure 4C), but the transient reduction in the number of erythrocytes was apparent in chickens infected with GX0101ΔMeq on days 7 p.i. (*P *< 0.05). Statistical analysis showed that there were no significant differences between chickens in the GX0101ΔMeq and control groups on days 14, 24, 31 and 42 p.i. (*P *> 0.05, Figure 4).

### Comparison of the immunosuppressive effects of two viruses on antibody responses

**Figure 5:**
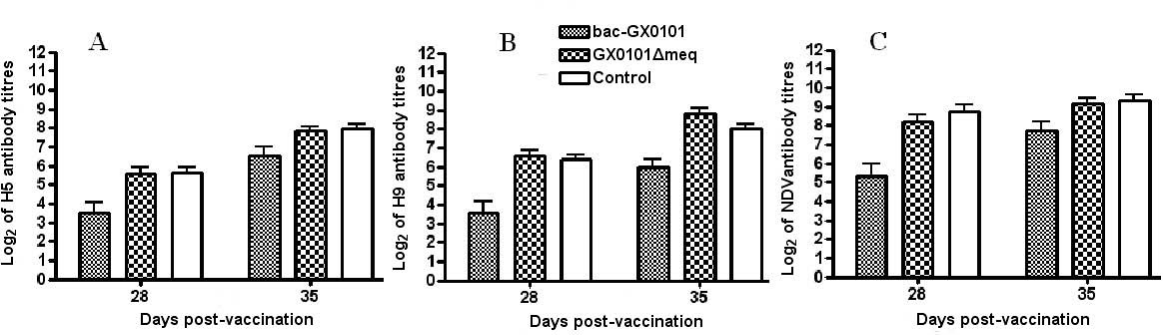
**Immunosuppressive effects of bac-GX0101 with X0101ΔMeq viruses on immune responses against NDV and AIV inactivated vaccines**. A: AIV-H5; B: AIV-H9; C: NDV. One-day-old chickens inoculated intra-abdominally with 1000 PFU of bac-GX0101, GX0101ΔMeq viruses or uninfected CEF cultures from each treatment were vaccinated with 0.3 ml inactive NDV (10^8 ^EID_50_/0.1 ml), AIV-H5(10^7.5 ^EID_50_/0.1 ml) and AIV-H9 (10^7.5 ^EID_50_/0.1 ml), at days 7 p.i., respectively. On days 28 and 35 post-vaccination, serum was collected to measure the HI antibody titers to NDV, AIV-H5 and AIV-H9. The means ± SD (n = 12) at each time point are shown. The bac-GX0101-infected chickens exhibited weaker humoral immune responses against the inactivated NDV and AIV vaccines compared to the control group on days 28 and 35 after immunization (*P *< 0.05). The GX0101ΔMeq-infected chickens showed similar antibody lever with the control group (*P *> 0.05).

To evaluate whether GX0101ΔMeq MDV had immunosuppressive effects on humoral immune responses, we compared the immunosuppressive effects of bac-GX0101 with GX0101ΔMeq viruses on immune responses against NDV and AIV inactivated vaccines. As expected, we found that the bac-GX0101-infected chickens exhibited weaker humoral immune responses against the inactivated NDV and AIV vaccines compared to the control group on days 28 and 35 after immunization (*P *< 0.05, Figure 5). The GX0101ΔMeq-infected chickens showed similar antibody lever with the control group (*P *> 0.05, Figure 5). These results indicated that GX0101ΔMeq had no immunosuppressive effects on humoral immune responses in chickens.

### Analysis of T cell subsets after inoculation with bac-GX0101 and GX0101ΔMeq

**Figure 6:**
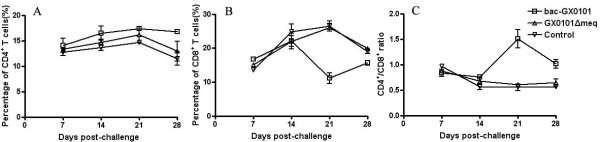
**Percentage of CD4+ and CD8+ T cells after inoculation of bac-GX0101 and GX0101ΔMeq (n = 4)**. One-day-old chickens inoculated intra-abdominally with 1000 PFU of bac-GX0101, GX0101ΔMeq viruses or uninfected CEF cultures, cell suspensions from chickens were obtained to analyze the percentage of CD8^+ ^lymphocytes in spleens at days 7, 14, 21 and 28 p.i. On days 21 and 28 p.i., the percentage of CD8^+ ^T cells was drastically reduced (*P *< 0.05) in bac-GX0101-infected chickens, however, the percentage of CD4^+ ^T cells was increased on days 14 and 21 p.i. (*P *> 0.05) and signifcantly increased on days 28 p.i. (*P *< 0.05) in bac-GX0101-infected chickens.

As shown in Figure 6 the percentage of CD8^+ ^T cells was drastically reduced in bac-GX0101-infected chickens on days 21 and 28 p.i. (*P *< 0.05), however, the percentage of CD4^+ ^T cells was increased on days 14 and 21 p.i. (*P *> 0.05) and significantly increased on days 28 p.i. (*P *< 0.05) in bac-GX0101-infected chickens. And the ratio of CD4^+ ^T cells to CD8^+ ^T cells in bac-GX0101-infected chickens significantly higher than the control group on days 21 and 28 p.i. (*P *< 0.05). However, the numbers of CD8^+^, CD4^+ ^T cells and CD4^+^/CD8^+ ^in GX0101ΔMeq-infected chickens were very similar to the control group (*P *> 0.05).

## Discussion

As known, MDV is one of the most contagious and highly oncogenic herpesviruses [1]. Apart from being an economically important disease affecting poultry health, MD has contributed significantly to our understanding of herpesvirus-associated oncogenicity [17]. Previous studies have revealed that the transcriptional regulator, Meq, is considered to be a major viral oncoprotein with a direct role in the induction of tumors. Recently, Reddy *et al*. [11] generated overlapping cosmid clones spanning the entire genome of a highly virulent oncogenic strain of MDV (Md5). Pathogenesis experiments showed that the rMd5ΔMeq virus was fully attenuated, and the Meq-null virus provided protection superior to CVI988/Rispens, the most efficacious vaccine presently available, following challenge with very virulent (rMd5) and very virulent plus (648A) MDV strains. However, little information is available regarding other biological characteristics of the Meq-null virus.

In the present study, we examined the oncogenic potential of GX0101ΔMeq. The results indicated that this mutant strain was not able to induce tumors, similar to the mutant rMD5ΔMeq [18]. It was reported that MDV infection greatly increased susceptibility to secondary challenge with pathogenic *Escherichia coli*, and reduced the antibody response to infectious bronchitis virus (IBV) vaccine as well [21,22]. In the current study, we compared the immunosuppressive effects of bac-GX0101 with GX0101ΔMeq viruses. Our data showed that the bac-GX0101-infected chickens exhibied weaker humoral immune responses against the inactivated NDV and AIV vaccines, consistent with the results of severe bursa and thymus lesions in bac-GX0101-infected chickens. However, there were no differences between the GX0101ΔMeq-infected chickens and the control chickens with respect to immunosuppressive effects. These results indicated that the Meq gene played an important role not only in tumor formation but also in inducing immunosuppressive affects in MDV-infected chickens.

The analysis of T cells subsets in chickens showed that chickens infected with bac-GX0101 exhibited not only suppressed humoral immune responses, but also down-regulation of the numbers of CD8^+ ^spleen cells. However, the numbers of CD8^+ ^spleen cells in GX0101ΔMeq-infected chickens was similar to those in the controls. These results suggest that the Meq gene is closely related to the down-regulation of CD8^+ ^spleen cells. It is well known that CD8^+ ^T cells play an important role in both protecting against MDV infection and tumor repression in chickens [23,24]. These findings suggest that the Meq gene may be involved in T cell immunosuppression in chickens infected with MDV.

It is possible that the reduced virus titers, which were due to the absence of Meq, are responsible for the lack of immunosuppression. In the previous studies, we inoculated SPF chickens with 100 PFU bac-GX0101 and 1000 PFU GX0101ΔMeq, respectively, and the virus titers of bac-GX0101 was lower than GX0101ΔMeq on days 7, 14, 21 and 28 p.i. However, the bac-GX0101, but not the GX0101ΔMeq, caused tumor and apparent suppression on humoral immune responses against the inactivated NDV and AIV vaccines in chickens (data not shown). These results indicated that the Meq gene played a direct role in immunosuppressive affects in MDV-infected chickens.

Early studies using mitogen stimulation assays suggested tumor cells were immunosuppressive, and addition of MDV-transformed lymphoblastoid cells to normal spleen cells inhibited the proliferative response to mitogens [25]. MDV induced transformation starts at 14 to 21 days p.i., and it appeared much earlier than immunosuppressive effects on the humoral immune responses against the inactivated NDV and AIV vaccines in chickens. These results indicated that the immunosuppressive effects might be partly due to Meq gene-associated tumor.

In the present study, our data showed that MDV induced immunosuppressive effects on the humoral immune responses against the inactivated NDV and AIV vaccines in chickens, and the immunosuppression appeared much earlier than the tumor formation. These results indicated that there was no relationship between the immunosuppressive effects and Meq gene-associated tumor. Therefore, the precise molecular mechanism by which the Meq gene induces immunosuppressive effects in chickens needs to be further studied.

## Conclusions

In this paper, we conclude that deletion of the Meq gene in MDV GX0101 contributes to a loss in pathogenicity and oncogenicity, and decreases immunosuppression in chickens. These results provide important initial experimental evidence for understanding the mechanisms of pathogenesis and immunosuppressive effects of MD.

## Methods

### Cell cultures and viruses

SPF chickens and chicken embryos for preparation of chicken embryo fibroblast (CEF) cultures were from SPAFAS Co. (Jinan, China; a joint venture with Charles River Laboratory, Wilmington, MA, USA). CEF cultures were used for virus propagation, virus reactivation assays and DNA transfections. SPF chickens were free of avian leukosis virus (ALV), reticuloendotheliosis virus (REV) and chicken infectious anemia virus (CAV).

### Construction of Meq-deleted GX0101 BAC clone

We cloned the full genome of GX0101 into a BAC and reconstituted the infectious virus, bac-GX0101 [19]. Gene disruptions of both copies of Meq in bac-GX0101 were performed according to a previous method [26]. Briefly, the mutagenesis strategy was to replace the targeted gene with a kanamycin resistance gene (kan^r^) by homologous recombination. Kan^r^, flanked by flp recognition target (FRT) sites from pKD13 [27], was amplified by polymerase chain reaction (PCR) using primers with 50 bp extensions that were homologous to the start and end of the coding sequence of the gene to be disrupted. The sequences of the primers used for deletion of Meq were: ΔMeq-F, 5'-AGA AAC ATG GGG CAT AGA CGA TGT GCT GCT GAG AGT CAC AAT GCG GAT CAc gtg tag gct gga gct gct tc-3', and ΔMeq-R 5'-CTT GCA GGT GTA TAC CAG GGA GAA GGC GGG CAC GGT ACA GGT GTA AAG AGc att ccg ggg atc cgt cga c-3', with MDV-specific sequences shown in capital letters.

The PCR products were used to transform the recipient EL250 cells harboring GX0101 BAC DNA by electroporation at 2000 V/100 Ω/25 μF [19], and recombinant clones were isolated as kanamycin-resistant colonies as previously described [28]. BAC DNA was isolated and examined for insertion of kan^r ^into the right locus using PCR. Once individual clones were examined and confirmed to lack spurious changes, kan^r ^was excised by induction of flp recombination by incubation in Luria-Bertani (LB) medium containing 0.02% arabinose for 12 h. Bacteria were diluted 1:1,000,000 in LB medium and plated onto LB agar containing 30μg/ml chloramphenicol. Individual colonies were re-streaked onto LB agar with chloramphenicol and LB agar with chloramphenicol and kanamycin to confirm that individual colonies were no longer kanamycin resistant. By using this technique, 100% of colonies screened were chloramphenicol resistant and kanamycin susceptible. This protocol was repeated for deletion of the second copy of Meq. Once both copies were deleted, recombinant virus, designated GX0101ΔMeq was reconstituted by transfecting CEF cultures with purified BAC DNA [29]. To identify the Meq deletion mutant, 100 plaque forming units (PFU) of bac-GX0101 and GX0101ΔMeq were inoculated into 6-well plates containing a monolayer of CEFs and incubated at 37°C/5% CO_2_. An IFA was carried out as described previously [30]. The mAb, specific for the MDV-unique protein pp38 (H19), was used at a working dilution of 1:300, and mouse serum against Meq was used at a working dilution of 1:200.

### In vitro replication

In vitro replication of mutant viruses was measured over time by counting the plaques on CEFs at various intervals. Briefly, 100 PFU of bac-GX0101 or GX0101ΔMeq were inoculated into 6-well plates and incubated at 37°C/5% CO_2_. At 0, 12, 24, 48, 72, 96, 120 and 144 h p.i., the plaques were counted.

### In vivo experiments

All experiments included three treatments (bac-GX0101, GX0101ΔMeq and control) in a completely randomized design. With the exception of experiment three, in each experiment, sixty male 1-day-old SPF birds were randomly divided into three equal groups (20 in each group) and reared separately in isolators with positive filtered air. When the birds were 1-day-old, in each group, chickens were inoculated intra-abdominally with 1000 PFU of bac-GX0101 or GX0101ΔMeq viruses, while control chickens were inoculated with uninfected CEF cultures.

#### Experiment 1

In vivo replication of GX0101Δmeq was measured by determining the viremia levels in chickens. In brief, blood samples in anticoagulants were collected from 6 randomly selected chickens from each group on days 7, 14 and 21 p.i., and buffy-coat cells were obtained by centrifugation. Lymphocytes from the buffy-coats were counted, diluted to 10^6 ^cells/ml and duplicated 35-mm plates of freshly seeded CEF monolayers infected with 10^6 ^lymphocytes for each chicken sample. To determine viremia levels, visible viral plaques were counted on days 6 p.i.

#### Experiment 2

To compare the pathogenic properties of bac-GX0101 with GX0101ΔMeq, after inoculation, chickens were evaluated daily for symptoms of MD and were euthanized and examined with gross lesions when they showed clinical evidence of MD. All surviving birds were sacrificed for necropsy after 13 weeks observation period to evaluate for gross lesions. Cumulative mortality and gross tumor rates were used for comparing the pathogenicity of each virus.

#### Experiment 3

To determine the effect of Meq on immune organs, 120 male 1-day-old SPF birds (40 in each group) were used in this experiment. At 14 days p.i., 20 chickens per group were used to evaluate thymic and bursal atrophy, and whole-bird body weights were measured prior to euthanasia. All thymus lobes and the bursa from each bird were weighed after collection, and the relative weight of the thymus and bursa to the whole body were determined [4]. Bursa and thymus of the surviving birds of each group were collected on days 28 p.i. and fixed in buffered 10% formalin, embedded in paraffin, and five micrometers-thick sections were stained with hematoxylin-eosin for histopathological evaluation.

#### Experiment 4

The effects of bac-GX0101 and GX0101ΔMeq on some blood parameters including erythrocytes, leukocytes and lymphocytes in peripheral blood were determined. At days 7, 14, 24, 31 and 42 p.i., 5 chickens were randomly selected from each treatment and blood was sampled into the anticoagulant, acid citrate dextrose and euthanized. Whole blood was used for hematology.

#### Experiment 5

To compare the immunosuppressive effects of the two viruses on the antibody response to vaccination, at days 7 p.i., all chickens from each treatment were vaccinated with 0.3 ml inactive NDV (10^8 ^EID_50_/0.1 ml), AIV-H5 (10^7.5 ^EID_50_/0.1 ml) and AIV-H9 (10^7.5 ^EID_50_/0.1 ml) with single dose, respectively. On days 28 and 35 post-vaccination, serum from 12 chickens of each group were randomly collected to measure the hemagglutination inhibition (HI) antibody titers to NDV, AIV-H5 and AIV-H9.

#### Experiment 6

In order to analyze the percentage of CD4^+ ^and CD8^+ ^lymphocytes in spleens, cell suspensions from 7, 14, 21 and 28 day post-inoculated chickens were obtained by disruption of spleens followed by Ficoll-Conray density gradient centrifugation to remove dead cells and red blood cells. Cell suspensions were stained with a FITC-conjugated anti-chicken CD4 mAb and an R-phycoerythrin (R-PE)-conjugated anti-chicken CD8α mAb (Southern Biotechnology Associate, Bimingham, Alabama, USA). Cells (1 × 10^6^) were incubated with the mAbs for 30 min at 4°C. After washing with PBS, the relative immunofluorescence of cells was analyzed by a flow cytometer (Guava EasyCyte Mini).

### Statistics analysis

Statistical analysis was performed with the SPSS statistical software package for Windows, version 13.0 (SPSS Inc., Chicago, IL, USA). Differences between groups were examined for statistical significance by a two-tailed Student *T*-test. A *P*-value less than 0.05 were considered statistically significant.

## Competing interests

The authors declare that they have no competing interests.

## Authors' contributions

YPL and AJS contributed to carry out most of the experiments and write the manuscript. HFZ and ZZC carried out study design, and revised the manuscript. SS and PZ conducted animal experiments and participated in data organization. And all authors have read and approved the final manuscript
